# A Phosphatidylinositol-3-Kinase-Dependent Signal Transition Regulates ARF1 and ARF6 during Fcγ Receptor-Mediated Phagocytosis

**DOI:** 10.1371/journal.pbio.0040162

**Published:** 2006-05-09

**Authors:** Peter Beemiller, Adam D Hoppe, Joel A Swanson

**Affiliations:** **1**Cellular and Molecular Biology Graduate Program, University of Michigan Medical School, Ann Arbor, Michigan, United States of America; **2**Department of Microbiology and Immunology, University of Michigan Medical School, Ann Arbor, Michigan, United States of America; University of California San FranciscoUnited States of America

## Abstract

Fcγ receptor (FcγR)–mediated phagocytosis of IgG-coated particles is regulated by 3′-phosphoinositides (3′PIs) and several classes of small GTPases, including ARF6 from the ADP Ribosylation Factor subfamily. The insensitivity of phagocytosis to brefeldin A (BFA), an inhibitor of certain ARF guanine nucleotide exchange factors (GEFs), previously indicated that ARF1 did not participate in phagocytosis. In this study, we show that ARF1 was activated during FcγR-mediated phagocytosis and that blocking normal ARF1 cycling inhibited phagosome closure. We examined the distributions and activation patterns of ARF6 and ARF1 during FcγR-mediated phagocytosis using fluorescence resonance energy transfer (FRET) stoichiometric microscopy of macrophages expressing CFP- or YFP-chimeras of ARF1, ARF6, and a GTP-ARF-binding protein domain. Both GTPases were activated by BFA-insensitive factors at sites of phagocytosis. ARF6 activation was restricted to the leading edge of the phagocytic cup, while ARF1 activation was delayed and delocalized over the phagosome. Phagocytic cups formed after inhibition of PI 3-kinase (PI-3K) contained persistently activated ARF6 and minimally activated ARF1. This indicates that a PI-3K-dependent signal transition defines the sequence of ARF GTPase activation during phagocytosis and that ARF6 and ARF1 coordinate different functions at the forming phagosome.

## Abstract 2

The authors use fluorescence resonance energy transfer (FRET) to track the localization of activated Arf1 and Arf6 GTPases, and show that both proteins are involved in Fcγ receptor-mediated phagocytosis.

## Introduction

Fcγ receptor (FcγR)-mediated phagocytosis by professional phagocytes requires the extension of a tightly apposed, actin- and membrane-rich pseudopod over the target particle. In spite of this commitment of plasma membrane, macrophages are able to engulf many particles without a concomitant loss in surface area [ 1]. The observation that inhibitors of type I phosphatidylinositol-3-kinase (PI-3K) prevent phagosome closure, but do not completely abolish actin polymerization and pseudopod extension, led to the suggestion that exocytic membrane insertion at sites of phagocytosis may be required for the complete extension of the phagosomal pseudopod [ 2]. The discovery that ADP ribosylation factor 6 (ARF6), a known regulator of plasma membrane trafficking, is required for FcγR-mediated phagocytosis, has underscored the link between phagocytosis and membrane delivery [ 3, 4].

Like all ARF GTP hydrolases (GTPases), ARF6 can regulate membrane trafficking events. In the context of phagocytosis, ARF6 is thought to mediate the focal exocytosis of vesicles that can fuse with the nascent phagosome [ 4]. ARF6 may also contribute to phagocytosis through its ability to activate lipid-modifying enzymes. GTP-bound ARF6 can activate phosphatidylinositol 4-phosphate 5-kinase α (PI4P-5Kα) during ruffling [ 5]. The product of PI4P-5Kα, phosphatidylinositol-4,5-bisphosphate [PI(4,5)P _2_], can facilitate actin polymerization through recruitment of WASP/N-WASP proteins [ 6]. Since PI(4,5)P _2_ is the substrate for type I PI-3K, ARF6 activation at the phagosome could contribute to the amplification of PI-3K signaling. ARF6 also activates phospholipase D (PLD) [ 7]. Activation of PLD is known to mediate a number of processes at the plasma membrane, including ruffling [ 8] and cell motility [ 9], in addition to FcγR-mediated phagocytosis [ 10, 11].

In contrast to ARF6, a role for ARF1 in phagocytosis has been discounted. ARF1 regulates a variety of intracellular trafficking compartments including the recently identified Golgi-localized, γ-ear-containing, ARF-binding protein (GGA) coats implicated in trans-Golgi network-endosome traffic [ 12]. The modular GGA-family proteins bind activated ARF GTPases through their GGA and TOM (GAT) domains [ 13]. However, in addition to its roles in Golgi trafficking, ARF1 can also serve as an effector of receptor-stimulated processes: ARF1 regulates the endocytosis of the μ-opioid receptor in a PLD-dependent manner [ 14], co-activates PLD in response to fMLP receptor ligation in neutrophils [ 15], and mediates the recruitment of paxillin to focal adhesions [ 16].

The function of ARF1 at the Golgi complex is dependent on Golgi-localized guanine nucleotide exchange factors (GEFs) that catalyze GDP release and incorporation of guanosine 5′-triphosphate (GTP) into the ARF structure. The binding of GTP stabilizes the interaction of ARF with membranes, allowing it to recruit effectors to sites of vesicle biogenesis [ 17, 18]. The Golgi-associated GEFs generally are inhibited by the fungal metabolite brefeldin A (BFA) [ 19, 20], distinguishing them from the BFA-insensitive cytohesin/ARNO and EFA families of GEFs [ 21]. Previous work showed that these BFA-resistant GEFs can regulate the activation of ARF6 [ 22, 23]. As ARF1 is typically thought to be regulated by the BFA-sensitive GEFs, the observation that phagocytosis proceeds in the presence of BFA suggested that ARF1 does not contribute to phagocytosis [ 3]. However, the assignment of GEFs to the regulation of different classes of ARF GTPases is complicated by the observation that, at least in vitro, cytohesin/ARNO family GEFs can catalyze nucleotide exchange on both ARF1 and ARF6 [ 22].

Immunofluorescence and biochemical methods have contributed greatly to our understanding of ARF GTPase functions in the cell. However, previous methods have not afforded a system for the in vivo observation of ARF activation dynamics. As a result, the precise location of ARF activation in the cell must be inferred from studies that rely on the localization of GTP-binding or GTP-hydrolysis mutant ARF proteins; these molecules may or may not accurately reflect the localization of endogenous GDP- or GTP-bound ARF proteins. Alternatively, biochemical methods have been developed to measure ARF activation but are unable to resolve where activation and deactivation occur in the cell. A complete understanding of the role of ARF family members in phagocytosis will require detailed analysis of the spatial and temporal profile of their activation during phagosome formation. Of particular interest is the sequence of activation of the various GTPases that coordinate the morphological changes required for phagocytosis.

In this study, we have used fluorescence microscopy to measure the localization and activation of ARF6 and ARF1 GTPases during phagocytosis. We show that constitutively active ARF1(Q71L) and the dominant-negative, GTP-binding-deficient ARF1(T31N) mutants partially inhibited phagocytosis when expressed in macrophages as cyan fluorescent protein (CFP) chimeras. Macrophages expressing the mutant ARF chimeras were able to initiate phagocytosis and began to extend pseudopods over opsonized target particles. However, the phagocytic cups in these macrophages frequently failed to close, a result similar to the inhibition of phagocytosis produced by the PI-3K inhibitor LY294002. Ratiometric fluorescence time-lapse microscopy indicated both ARF6-YFP and ARF1-YFP chimeras localized to sites of FcγR-mediated phagocytosis. To quantify the activation of these ARF molecules in vivo, we used a portion of the GGA1 GAT domain, the α-helical N-GAT, to develop an assay based on fluorescence resonance energy transfer (FRET) stoichiometry [ 24]. Using this FRET microscopy method, we observed that both ARF6 and ARF1 were activated during the extension of phagosomal pseudopods. Activation of ARF6 was concentrated at the tip of extending pseudopods, while ARF1 was active throughout the forming phagosome. ARF6 activation was independent of PI-3K activity, but deactivation of ARF6 required PI-3K function. In contrast, ARF1 activation at the phagosome was blocked by PI-3K inhibition. Furthermore, we found that the phagosome-localized activation of ARF1 was unaffected by BFA, indicating ARF1 may be regulated by cytohesin/ARNO family GEFs in response to FcγR ligation. The distinct responses of ARF6 and ARF1 activation to PI-3K inhibition suggests that these ARF GTPases regulate distinct functions during FcγR-mediated phagocytosis.

## Results

### FRET-Based Measurement of ARF GTPase Activation

To localize ARF activation inside live cells, we developed a FRET microscopic method based on the interaction between GTP-bound ARFs and GGA-family protein GAT domains. The binding of GAT domains to activated ARFs has been used previously for biochemical measurements of ARF6 activation [ 4, 9]. To create a marker that distributes in the cell solely in an ARF-GTP-dependent manner, we created a fusion of yellow fluorescent protein (YFP) to 45 amino acids from the N-terminus of the GGA1 GAT domain (NGAT). This fragment of the GAT domain incorporates all the amino acid residues known to contribute to the binding of ARF-GTP [ 25, 26, 27] but lacks the C-terminal segment that interacts with the Rab5 GTPase activating protein (GAP) Rabaptin-5 [ 28]. The affinity of this NGAT domain for ARF1-GTP was reported to be comparable to the affinity of the full-length GGA1 GAT domain [ 29]. Despite its ability to bind to ARF-GTP, expression of YFP-NGAT was not toxic to macrophages, nor did it appear to inhibit phagocytosis (see below). In contrast, we were not able to express at high levels in macrophages a YFP-NGAT construct derived from the human GGA3 cDNA; this construct inhibited phagocytosis even at low expression levels. We attribute these different results for the two chimeras to different affinities for ARF-GTP. The apparently lower affinity of the GGA1-derived YFP-NGAT allowed it to act as a reporter of ARF activation without inhibiting the RAW264.7 cells' phagocytic functions.

When co-expressed in cells, the binding of YFP-NGAT to GTP-bound ARF-CFP chimeras could be detected using FRET microscopy. Following collection of I _A_, I _D_, and I _F_ images, image processing using FRET stoichiometry generated two images that reflect the GTP-dependent interaction of ARF-CFP and YFP-NGAT chimeras [ 30]: E _A_, which is proportional to the fraction of YFP acceptors in complex with CFP donors, and E _D_, which is proportional to the fraction of CFP donors bound to YFP acceptors. Increases and decreases in the E _A_ and E _D_ images reflect changes in the amount of ARF-CFP complexed with YFP-NGAT. Additionally, from the component fluorescence images, FRET stoichiometry determines R _M_: the molar ratio of all YFP acceptors (free and complexed) to all CFP donor molecules.

To evaluate the ability of this method to detect ARF-GTP, RAW264.7 macrophage-like cells co-expressing ARF1-CFP and YFP-NGAT were used to visualize activated ARF1 at the Golgi apparatus and to measure ARF1 deactivation in response to BFA. On the molecular level, BFA binds an ARF–GDP–GEF intermediate complex preventing release of the GDP nucleotide [ 31]. At the organellar level, this results in the collapse of the Golgi stacks [ 32].

**Figure 1:**
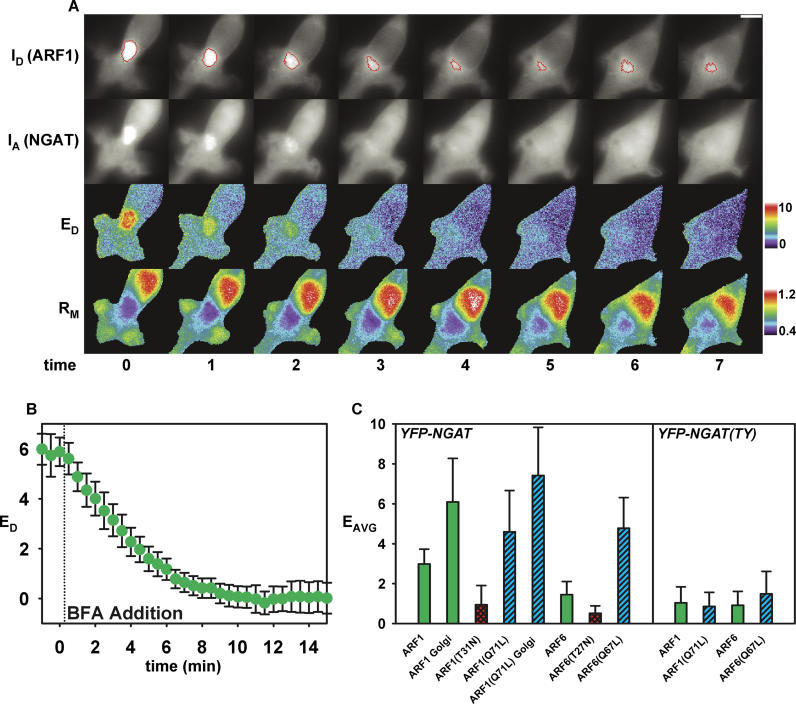
Measurement of ARF Activation Using FRET Stoichiometry (A) Time points (in minutes) are relative to the addition of 5 μm BFA. ARF1-CFP and YFP-NGAT were enriched at the Golgi complex in transfected RAW264.7 macrophages. BFA rapidly disintegrated the Golgi network (shrinking region outlined in red in the ARF1-CFP panels), and the fluorescent markers redistributed to the cytosol. Color bars to the right of the E _D_ and R _M_ panels indicate the magnitude of E _D_ expressed as a percent, and the molar ratio of YFP-NGAT/ARF1-CFP, respectively. The values to the right of the color bar indicate the values of the pixel colors at the ends of the scale. For each image series and video presented in this work, all images within a single time series are identically scaled and directly comparable. Note that in the region of the Golgi apparatus, ARF1-CFP was present at an ˜2.5-fold molar excess relative to YFP-NGAT. YFP-NGAT localization to the Golgi apparatus was mediated by ARF1-GTP binding as indicated by the high E _D_ values at the Golgi. The FRET-based detection of ARF1-activation rapidly decreased after the addition of BFA; nearly all ARF1 activation was blocked within ˜5 minutes of the addition of BFA. The scale bar located in the upper right of the CFP time series is 6 μm. (B) The decrease in E _D_ signals at the Golgi apparatus corresponding to loss of active ARF1 was quantified. An inclusive threshold was used to generate regions (as shown in the ARF1-CFP panels above) and to mask the E _D_ image to measure the Golgi-associated activation of ARF1 after BFA addition. Following the addition of BFA (dotted line), ARF1-GTP levels at the Golgi immediately began to decrease; *n* = 6 cells. Error bars represent ± the standard error of the mean (SEM). (C) FRET values expressed as the average of the percent E _A_ and E _D_ signals were measured in macrophages expressing the indicated FRET partners. The dominant-negative ARF-CFP chimeras produced low, but detectable, FRET signals when co-expressed with YFP-NGAT. Conversely, co-expression of the constitutively active ARF1(Q71L)-CFP or ARF6(Q67L)-CFP chimeras with YFP-NGAT produced elevated FRET signals compared with wild-type ARF-CFP chimeras. ARF1(Q71L)-CFP and YFP-NGAT generated slightly elevated FRET signals at the Golgi apparatus. The bar plots to the right of the black line are FRET signals measured when the listed ARF-CFP chimera was co-expressed with YFP-NGAT(A193T, N194Y), an NGAT molecule with reduced ARF-GTP binding affinity. Neither the wild-type nor constitutively activated ARF-CFP chimeras generated high FRET signals when co-expressed with YFP-NGAT(A193T, N194Y). Measurements for each FRET pair are the average from at least 25 cells, and error bars represent plus/minus the standard deviation.

Prior to the addition of BFA, ARF1-CFP and YFP-NGAT colocalized to the juxtanuclear region but were also present throughout the cytoplasm ( Figure 1A). ARF1-CFP accumulation resulted in a molar excess of ARF1-CFP donors to YFP-NGAT acceptors at the Golgi complex ( Figure 1A, R _M_ panels). This site of ARF1-CFP and YFP-NGAT colocalization corresponded to the region of high E _D_ indicating binding of YFP-NGAT to activated ARF1-CFP ( Figure 1A, time 0 E _D_ image). Following the addition of 5 μM BFA to macrophages, both ARF1-CFP and YFP-NGAT redistributed to the cytoplasm ( Figure 1A). YFP-NGAT completely dispersed within ˜5 min of the addition of BFA. ARF1-CFP was also rapidly released from the Golgi complex but remained slightly enriched in the juxtanuclear region for ˜15 min after BFA addition. Similarly, both the E _D_ ( Figure 1A and 1B) and E _A_ (unpublished data) signals at the Golgi apparatus rapidly decreased after the delivery of BFA. With activation of ARF1 arrested by BFA, stimulation of GTP hydrolysis by endogenous GAPs led to the rapid deactivation of all ARF1-CFP at the Golgi complex within ˜7 min. The kinetics observed for BFA-induced ARF1-CFP and YFP-NGAT redistribution and ARF1 deactivation were consistent with a previous study of the effects of BFA on the localization of ARF1-CFP and YFP-GAT chimeras [ 26].

When YFP-NGAT was co-expressed in RAW264.7 cells with ARF1(T31N)-CFP or ARF6(T27N)-CFP, mutations known to reduce the affinity of the ARF GTPases for GTP [ 33, 34], cells exhibited reduced FRET signals ( Figure 1C). Similarly, much lower FRET signals were measured from cells expressing wild-type ARF-CFP chimeras with YFP-NGAT(A193T, N194Y). This mutant NGAT construct is expected to have significantly reduced affinity for ARF-GTP based on previous studies of the ARF-GAT domain interaction [ 25– 27]. In contrast, the constitutively active mutants ARF1(Q71L)-CFP and ARF6(Q67L)-CFP both produced elevated FRET signals when co-expressed with YFP-NGAT in macrophages ( Figure 1C); however, increased FRET signals were not seen when the GTP hydrolysis-deficient ARF molecules were co-expressed with the YFP-NGAT(A193T, N194Y) molecule. The average FRET values measured from cells expressing the constitutively active or constitutively inactive ARF-CFP chimeras were significantly different from the corresponding wild-type CFP chimeras (Mann-Whitney U test, *p* < 0.001). Likewise, the FRET values obtained from cells expressing the mutant YFP-NGAT chimera with wild-type or constitutively active ARF-CFP were significantly different from the wild-type YFP-NGAT controls ( *p* < 0.001). The average E _A_, E _D_, E _AVG_, and R _M_ values of the samples used to produce Figure 1C can be found in Table S1. The ARF-CFP chimeras never indicated FRET when co-expressed with free YFP (unpublished data). These results indicate that the FRET signals measured by FRET stoichiometry are dependent on the binding of activated ARF-CFP chimeras to the YFP-NGAT molecule, and that FRET stoichiometry is capable of measuring ARF activation dynamics in living cells.

### Localization of ARF6, ARF1, and NGAT during FcγR-Phagocytosis

Time-lapse ratiometric microscopy of RAW264.7 cells engaged in phagocytosis of IgG-opsonized erythrocytes, and expressing CFP and ARF6-YFP or ARF1-YFP, was used to localize the GTP-binding proteins to sites of phagocytosis. R _M_ images calculated from cells expressing YFP chimeras and CFP represent the molar ratio of YFP chimera relative to soluble CFP at each pixel in the image. As such, R _M_ images generated from cells co-expressing YFP chimeras with CFP measure the specific recruitment of YFP chimeras to sites in the cell independent of path-length contributions to image intensities.

**Figure 2:**
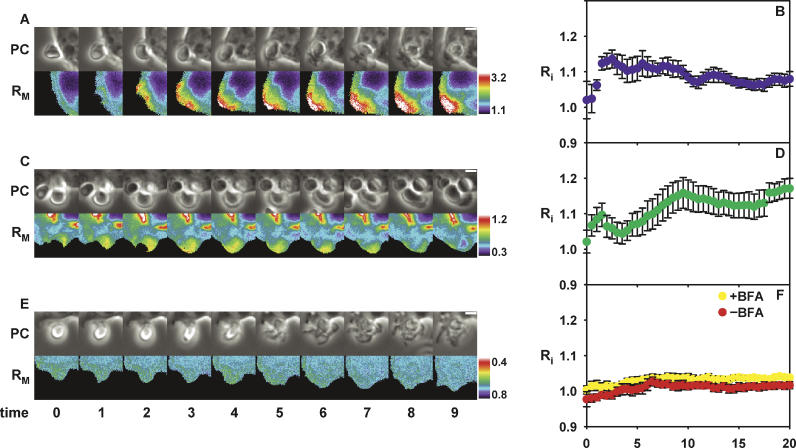
Ratiometric Microscopy of Arf6-YFP, ARF1-YFP, and YFP-NGAT at the Phagosome Ratiometric fluorescence microscopy of macrophages expressing ARF1-YFP, ARF6-YFP, or YFP-NGAT chimeras with soluble CFP during phagocytosis. (A, C, and E) Phase contrast (PC) and ratiometric images of macrophages during the phagocytosis of IgG-opsonized erythrocytes. Scale bars are 3 μm. Color bars indicate the molar ratio of YFP-chimera/CFP in the adjacent R _M_ images. (B, D, and F) Plots of the average recruitment index (R _i_) for temporally aligned phagocytic events. Error bars correspond to ± SEM. (A) ARF6-YFP rapidly accumulated at sites of phagocytosis; the region of high ARF6-YFP concentration advanced over the particle during pseudopod extension. (B) Analysis of multiple phagosomes indicated that the level of ARF6-YFP on the phagosome peaked during the first 2.5 min of engulfment. Following this initial rise, localization to the phagosome gradually decreased but ARF6-YFP remained associated with the phagosome well after closure at 7–8 min; *n* = 10 phagosomes. (C) ARF1-YFP localization to the phagosome was less pronounced than ARF6-YFP. ARF1-YFP generally accumulated over the entire phagosome and was also slightly enriched in membrane ruffles formed following closure. (D) Averaged particle-tracking results indicated ARF1-YFP association increased transiently during the first 2 min of phagosome formation, then increased further after 5 min. ARF1-YFP levels at the phagosome plateaued shortly after closure and remained elevated over the course of observation; *n* = 11 phagosomes. (E) The concentration of YFP-NGAT did not increase at sites of phagocytosis relative to the cytosol, indicating the level of activated ARF GTPases at the phagosome was too low to detect using YFP-NGAT ratiometric imaging. (F) Aggregate particle-tracking data never indicated recruitment of YFP-NGAT to phagosomes in the presence or absence of the ARF GEF inhibitor BFA. The time course of phagosomes formed without BFA pretreatment is the average of seven phagosomes. The plot of phagosomes formed in the presence of BFA was generated from ten phagocytic events.

Based on the average length of time required to complete phagocytosis, expression of ARF6-YFP did not inhibit phagocytosis. During phagocytosis, ARF6-YFP was recruited to the leading edges of extending pseudopods ( Figure 2A). The highest ARF6-YFP enrichment occurred in the front of the advancing phagocytic cups, and ARF6-YFP was prominently enriched in post-phagocytic ruffles after engulfment of the opsonized erythrocytes. Following closure of the phagosome at ˜7–8 min after erythrocyte binding, ARF6-YFP remained associated with the phagosome at moderate levels relative to the surrounding cytoplasm ( Figure 2A and 2B). Analysis of multiple phagosomes showed that ARF6-YFP localization to the phagosome increased rapidly during the extension of phagosome pseudopods, peaked within ˜2.5 min of the initiation of phagocytosis, then gradually diminished ( Figure 2B).

Unlike ARF6, ARF1-YFP recruitment did not consistently associate with a particular subregion of the phagosome ( Figure 2C). Maximum recruitment of ARF1-YFP to the phagosome occurred later than the peak in ARF6-YFP localization ( Figure 2B and 2D). Based on the aggregate analysis of independent phagosomes, recruitment of ARF1-YFP appeared biphasic: ARF1-YFP was transiently recruited upon the initiation of pseudopod extension but was recruited to greater extent as phagosomes neared closure. ARF1 recruitment to the phagosome plateaued shortly after sealing of the phagosome (˜10 min) and remained elevated over the course of observation ( Figure 2D).

We next attempted to use the YFP-NGAT fusion protein to measure the distribution of endogenous activated ARF proteins at the phagosome. The YFP-NGAT chimera was distinctly enriched at juxtanuclear sites indicating binding to endogenous, activated ARFs in the Golgi network ( Figure 1B). However, during FcγR-mediated phagocytosis, no significant recruitment of the YFP-NGAT chimera to phagosomes was observed ( Figure 2E and 2F). Analysis of these phagosomes suggested that either limited or no activation of endogenous ARF proteins occurred during phagocytosis, or the affinity of the YFP-NGAT chimera for activated ARF proteins did not allow it to compete effectively with endogenous ARF-GTP-binding proteins. Pretreatment of macrophages with BFA prior to microscopic observation redistributed the YFP-NGAT evenly throughout the cell; however, YFP-NGAT still did not appear to localize to sites of phagocytosis in these cells ( Figure 2F). The fact that YFP-NGAT was not recruited to phagosomes, combined with the observation that YFP-NGAT did accumulate at the Golgi complex, indicates the total level of ARF-GTP at the phagosome was much lower than the amount of activated ARF in the Golgi compartment.

### ARF6 and ARF1 Activation at Sites of Phagocytosis

To measure the GTP-binding state of ARF6 and ARF1 at phagosomes, ARF6-CFP or ARF1-CFP chimeras were co-expressed with YFP-NGAT chimeras in RAW264.7 cells and observed during FcγR-mediated phagocytosis. In macrophages co-transfected with ARF6-CFP and YFP-NGAT, activated ARF6-CFP was observed in ruffles ( Video S1), consistent with previous work that demonstrated ARF6 and ARNO3 translocate to sites of ruffling [ 23]. Although ruffles and phagosomes typically displayed comparable E _D_ values, ARF6 accumulated to a greater extent at phagosomes than in ruffles ( Video S1). This indicates that while the fraction of ARF6 bound to GTP was approximately equal in ruffles and phagosomes, the total amount of ARF6 activated in response to FcγR-ligation was higher.

**Figure 3:**
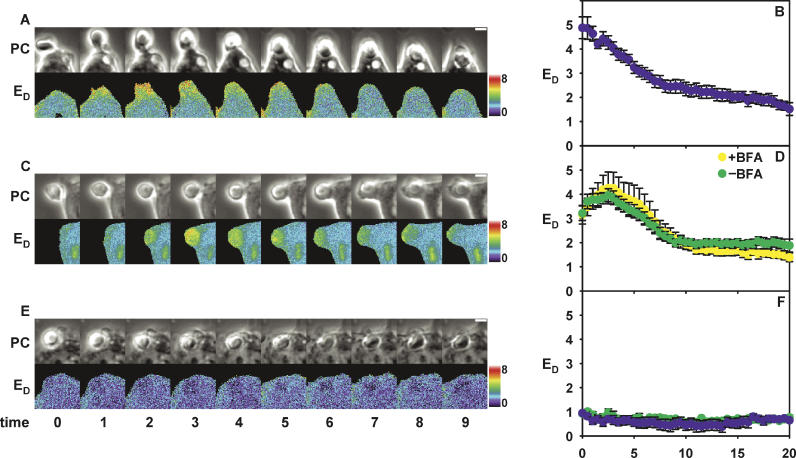
Measurement of ARF6-CFP and ARF1-CFP Activation at Phagosomes FRET microscopic measurement of ARF6-CFP and ARF1-CFP activation during phagocytosis. (A, C, and E) Phase contrast and E _D_ images of phagocytic events from macrophages co-expressing ARF6-CFP and YFP-NGAT, ARF1-CFP and YFP-NGAT, or ARF6-CFP and YFP-mutant NGAT. Scale bars in the upper right of the phase contrast series are 3 μm. (B, D, and F) Plots of E _D_ against phagosome progress for the corresponding pairs in (A), (C), and (E). Error bars indicate ± SEM. (A) ARF6-CFP activation peaked shortly after particle binding. Activated molecules were concentrated at the leading edge of the phagosome with lower levels of ARF6-GTP in the region trailing the advancing edge of the pseudopod. (B) Based on compiled particle-tracking results, maximum ARF6-CFP activation occurred shortly after the initiation of phagocytosis, and most of the ARF6-CFP was deactivated by the time of closure (˜7–8 min after the initiation of phagocytosis); *n* = 11 phagocytic events. (C) The peak in ARF1-CFP activation was slightly delayed relative to ARF6-CFP, and ARF1-GTP was not restricted to a subregion of the phagosome. ARF1-GTP levels began to decrease as the closure phase began. The persistent Golgi-associated FRET signal indicated ARF1-GTP levels at the Golgi did not fluctuate during phagocytosis. (D) Analysis of multiple phagosomes indicated ARF1-CFP was also almost entirely deactivated by the time of phagosome closure; *n* =14. The presence of BFA did not inhibit the activation of ARF1-CFP at sites of phagocytosis, suggesting ARF1 is activated by BFA-insensitive GEFs during phagocytosis. 14 phagocytic events that occurred in the presence of 5 μM BFA were analyzed by particle-tracking analysis. (E) ARF6-CFP produced very low E _D_ values at sites of phagocytosis when co-expressed with YFP-NGAT(A193T, N194Y). (F) Multiple phagosomes from macrophages co-expressing the binding-deficient YFP-NGAT(A193T, N194Y) with ARF6-CFP (blue) or ARF1-CFP (green) never demonstrated significant FRET signals. ARF1 results are the average of 15 phagosomes; ARF6 results represent eight phagosomes.

In our analysis of 11 phagosomes, ARF6 was activated during all phagocytic events, although the magnitude of the activation varied significantly between phagosomes. FRET microscopy indicated ARF6 activation immediately upon binding of opsonized particles to the surfaces of macrophages ( Figure 3A). During extension of the phagocytic cup, activated ARF6-CFP was concentrated at the advancing edge of the pseudopod with lower E _D_ values over the base of the phagosome ( Figure 3A and Video S1). Based on particle-tracking analysis of multiple phagosomes, the maximum activation of ARF6-CFP occurred within the first two minutes of phagocytosis and most of the ARF6-CFP on the phagosomal membrane was deactivated by the time of phagosome closure (˜7–8 min) ( Figure 3A and 3B and Video S1). It should be noted that to make quantitative comparisons of independent phagocytic events, the particle-tracking analysis discards much of the spatial resolution provided by the microscope. Comparing the averaged tracking analysis results to the time series of ARF6 activation (compare Figure 3B and 3C) showed that the immediate decrease in E _D_ signal following erythrocyte binding seen in the tracking results was not reflected in the time series of E _D_ images. This is the result of the advance of an increasing portion of the pseudopod into the measurement region; as the measurement region encompasses a larger area of the macrophage cytoplasm, the FRET signal at the advancing tip is averaged with the trailing region of low FRET. Similar patterns and profiles were reported for Cdc42 activation [ 30].

In contrast to ARF6-CFP, ARF1-CFP activation generally increased during pseudopod extension, but ARF1-CFP was also mostly deactivated by the time of phagosome closure ( Figure 3C and 3D and Video S2). The timing of peak ARF1-CFP activation varied from phagosome to phagosome with peaks at 0 min (one phagocytic event), 0.5 min (two events), 1 min (one event), 1.5 min (one event), 2.5 min (three events), 3.5 min (one event), and 4 min (one event). The region of high E _D_ was not restricted to a subregion of the nascent phagosome but rather was generally distributed over most of the particle.

To identify the family of ARF GEFs that catalyzed the activation of ARF1-CFP at sites of phagocytosis, macrophages co-expressing ARF1-CFP and YFP-NGAT were pretreated with BFA before microscopic observation. Prior to phagocytosis, these cells exhibited uniform molar ratios of YFP-NGAT/ARF1-CFP throughout their cytoplasm, and E _D_ values in these cells were ˜0. Similar to phagocytic events that occurred in the absence of BFA, upon particle binding ARF1-CFP activation increased, peaking within 4 min of the start of phagocytosis. Deactivation occurred as the phagosome closed over the target ( Figure 3D and Video S3). Based on the tracking of multiple phagosomes, the magnitude and timing of ARF1 activation and deactivation at sites of phagocytosis were not affected by the presence of BFA ( Figure 3D and Video S3). ARF6 activation also was not affected by the presence of BFA (unpublished data). The BFA-resistance of phagosome-associated ARF1-CFP activation implicates a member of the cytohesin/ARNO family of GEFs in the function of ARF1 during phagocytosis.

When ARF6-CFP or ARF1-CFP were co-expressed with a mutant YFP-NGAT(A193T, N194Y) designed to block binding to activated ARF proteins, macrophages demonstrated low FRET signals of 0%–1% throughout their cytoplasm with no increase observed at the phagosome ( Figure 3E and 3F). The low E _D_ signals in these control cells indicated that FRET was dependent on the binding of YFP-NGAT to activated ARF GTPases.

### ARF1 Activation and ARF6 Deactivation at Sites of Phagocytosis Required PI-3K Activity

**Figure 4:**
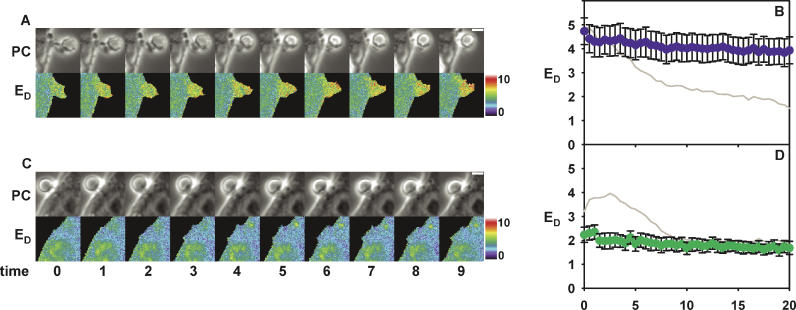
Activation of ARF6 and ARF1 during Phagocytosis in Macrophages Pretreated with LY294002 Macrophages co-expressing ARF6-CFP or ARF1-CFP with YFP-NGAT were treated with LY294002 for 30 min to inhibit PI-3K. (A) Phase contrast and E _D_ images from an RAW264.7 macrophage expressing ARF6-CFP and YFP-NGAT. Binding of the IgG-opsonized erythrocyte led to activation of ARF6-CFP, but the phagosome never closed, and ARF6-CFP was not deactivated. (B) The particle-tracking analysis for ten phagosomes from cells co-expressing ARF6-CFP and YFP-NGAT indicated that the magnitude of ARF6 activation in response to FcγR ligation was not reduced, but ARF6 deactivation could not proceed in the presence of PI-3K inhibitor. (C) Phase contrast and E _D_ images from a macrophage expressing ARF1-CFP and YFP-NGAT did not demonstrate a phagosome-localized activation of ARF1 in the presence of LY294002. However, ARF1-CFP activation at the Golgi apparatus appeared to be unaffected (crescent-shaped region of high E _D_ near the nucleus). (D) Measurement of ARF1 activation in LY294002 by particle-tracking analysis of nine phagosomes showed that a consistent but low level of ARF1 was bound to GTP at phagocytic cups; these macrophages did not display the transient, localized activation of ARF1 seen in control cells not treated with LY294002. Gray lines in (B) and (D) are the activation profiles in the absence of LY294002 for ARF6 and ARF1, respectively, taken from Figure 3B and 3D. Error bars represent ± SEM. Scale bars in (A) and (C) are 3 μm.

To determine whether the activation of ARF1 and ARF6 at phagosomes was regulated by PI-3K, macrophages co-expressing YFP-NGAT with ARF1-CFP or ARF6-CFP were exposed to LY294002 to inhibit PI-3K activity prior to microscopic observation. Treatment with LY294002 inhibits the production of phosphatidylinositol-3,4,5-trisphosphate [PI(3,4,5)P _3_], blocking phagocytosis at a stage distal to receptor ligation and phagocytic cup formation [ 35, 36]. Despite the presence of LY294002, formation of the phagocytic cup was accompanied by activation of ARF6-CFP ( Figure 4A and Video S4). Particle-tracking analysis indicated ARF6-CFP was still activated immediately upon binding and remained activated over the course of observation ( Figure 4B). Despite the activation of ARF6, macrophages were not able to complete phagocytosis in the presence of PI-3K inhibitor. This result suggests that ARF6 deactivation at the phagosome, rather than activation, is coupled to PI-3K activity.

In contrast, ARF1-CFP activation at phagocytic cups did not increase in the presence of PI-3K inhibitor ( Figure 4C and Video S5). Macrophages challenged with LY294002 still demonstrated low levels of ARF1-GTP at phagocytic cups but did not produce the transient increase in ARF1 activation observed during phagocytosis in the absence of LY294002 (compare Figures 3D and 4D). Multiple phagosomes never indicated an increase in ARF1-CFP activation at sites of phagocytosis, although Golgi-associated ARF1-CFP activation persisted in the presence of LY294002 ( Figure 4C). Taken together, these results indicate ARF1 activation at the phagosome was dependent on a PI-3K-regulated activity, and that LY294002 arrested phagocytosis at a point preceding ARF6 deactivation.

### ARF1 GTPase Mutants Block Phagocytosis after the Initiation of Pseudopod Extension

**Figure 5:**
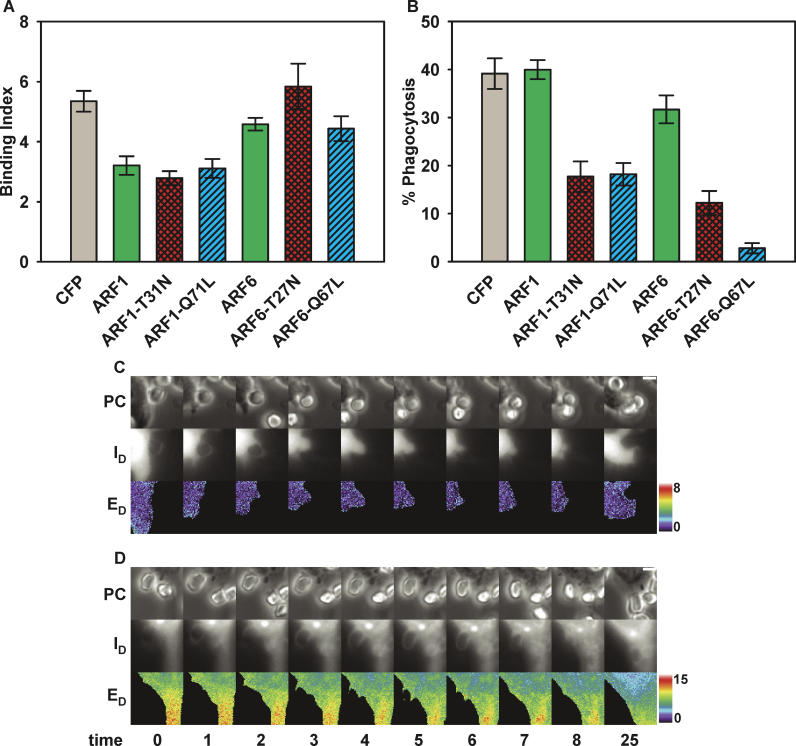
Effect of ARF1 and ARF6 Cycling Mutants on the Phagocytic Efficiciency of Macrophages and Imaging of ARF1(T31N)- and ARF1(Q71L)-Arrested Phagosomes (A) The effects of expression of the ARF-CFP chimeras on the binding of opsonized target particles. The ARF1-CFP chimeras generally reduced the binding index of macrophages, while the ARF6-CFP chimeras did not affect target particle binding. (B) Phagocytic indexes were measured for macrophages expressing CFP fusions of ARF1, ARF1(T31N), ARF1(Q71L), ARF6, ARF6(T27N), or ARF6(Q67L). The number of internalized and bound, uninternalized erythrocytes were counted for macrophages expressing the various constructs. Although ARF1-CFP did not reduce phagocytosis, expression of ARF1(T31N)-CFP or ARF1(Q71L)-CFP led to a reduction in phagocytic efficiency. Similarly, ARF6-CFP, in its wild-type form, produced a small inhibition of phagocytosis, but the constitutively activated and dominant-negative forms of ARF6-CFP impaired phagocytosis to a much greater degree. For all constructs except ARF6(Q67L)-CFP, results are the average of at least two experiments in which three coverslips were assayed. At least 25 macrophages were counted per coverslip. The ARF6(Q67L)-CFP results are from 31 macrophages counted on six coverslips. The error bars in (A) and (B) represent ± SEM. (C and D) Time-lapse fluorescence microscopy from macrophages expressing YFP-NGAT and ARF1(T31N)-CFP or ARF1(Q71L)-CFP. Note that the last image in the series was taken 25 min after the initiation of phagocytosis. Control RAW264.7 macrophages typically required 6–8 min to complete phagocytosis from the onset of pseudopod extension. The scale bars in the far-right phase contrast panels are 3 μm. (C) In macrophages expressing ARF1(T31N)-CFP, phagocytic cups formed, but the macrophages failed to close the phagosome. (D) Expression of ARF1(Q71L)-CFP also prevented phagosome closure. The phagocytic cup is visible in the fluorescence image.

To test the effects of the constitutively active and dominant-negative ARF-CFP molecules on phagocytosis, we measured phagocytic efficiencies for cells expressing CFP, wild-type ARF-CFP, or cycling mutant ARF-CFP chimeras. Expression of ARF1-CFP reduced the binding index of macrophages by 40% compared with macrophages expressing CFP, and expression of the cycling mutant ARF1-CFP chimeras reduced binding indexes by ˜40%–50% ( Figure 5A). Despite the reduction in the number of erythrocytes bound, macrophages expressing ARF1-CFP were able to internalize bound, opsonized erythrocytes with the same efficiency as macrophages expressing CFP. However, expression of ARF1(T31N)-CFP or ARF1(Q71L)-CFP reduced the phagocytic efficiency of macrophages by approximately 2-fold compared with cells expressing CFP ( Figure 5B). Student's *t*-tests indicated that the phagocytic efficiencies of the macrophages expressing the ARF1-CFP mutants were significantly different from the macrophages expressing CFP ( *p* ≤ 0.01). Similarly, we found that while wild-type ARF6-CFP produced a 20% decrease in phagocytic efficiency when compared with macrophages expressing CFP, the cycling mutants ARF6(T27N)-CFP and ARF6(Q67L)-CFP sharply reduced the phagocytic efficiency of the RAW264.7 macrophages by at least 70% ( Figure 5B). None of the ARF6 chimeras had a large effect on the binding indexes of macrophages ( Figure 5A). These results demonstrate that the closure of FcγR-mediated phagosomes is dependent on the normal cycling of both the ARF1 and ARF6 GTPases.

To determine the stage at which mutant ARF1 arrested phagocytosis, time-lapse fluorescence microscopy movies were acquired using RAW264.7 macrophages expressing YFP-NGAT and ARF1(T31N)-CFP or ARF1(Q71L)-CFP. In macrophages expressing ARF1(T31N)-CFP and YFP-NGAT, neither marker accumulated in the juxtanuclear region. In contrast, expression of ARF1(Q71L)-CFP did not inhibit the localization of YFP-NGAT to the juxtanuclear Golgi region. However, the Golgi compartment did frequently appear more fragmented in macrophages expressing the constitutively active ARF1 chimera. Macrophages expressing either of these mutant ARF-CFP chimeras were able to initiate phagocytosis. However, phagocytic cups formed in macrophages expressing ARF1(T31N)-CFP or ARF1(Q71L)-CFP did not progress past pseudopod extension ( Figure 5C and 5D). The macrophages expressing ARF1(T31N)-CFP and YFP-NGAT never exhibited FRET. Macrophages expressing ARF1(Q71L)-CFP and YFP-NGAT did display FRET; however, the FRET signals observed were not dynamic ( Figure 5D). The phagocytic cups formed in these macrophages resemble the cups formed when PI-3K activity was inhibited by pretreatment of macrophages with LY294002.

## Discussion

FRET microscopy revealed that PI-3K activity establishes a molecular switch that controls the activation dynamics of ARF GTPases during FcγR-mediated phagocytosis. This signal transition comprises a temporal boundary identified by the deactivation of ARF6 and the activation of ARF1. This PI-3K-dependent regulatory mechanism defines a sequence of signaling events during phagocytosis. While this progression occurs smoothly as the phagocyte engulfs the particle, the signal transition initiated by 3′PIs distinguishes early and late phagosomal activites regulated by different GTPases from the ARF, and perhaps Rho, family.

It was previously demonstrated that PI-3K is recruited to phagosomes, and that PI(3,4,5)P _3_ accumulates at phagosomes, reaching a plateau shortly before closure, then is cleared from phagosome [ 37]. These observations can be aligned with the signal transition model presented here. As phagocytosis commences, ARF6 is activated independently of PI(3,4,5)P _3_ production. However, as PI-3K products accumulate in the phagocytic membrane, GAP activities are recruited to deactivate ARF6, while GEFs simultaneously translocate to the phagosome to activate ARF1. As PI(3,4,5)P _3_ is cleared from the closing phagosome, ARF1 GEF activity also decreases, and all ARF GTPases are deactivated.

ARF6 was rapidly activated upon FcγR ligation and primarily active during pseudopod extension. ARF6 activation was focused at the advancing edge of pseudopods, where it may regulate the exocytosis of membrane to complete particle engulfment [ 2, 4]. The decreasing E _D_ values seen at phagocytic cups during closure likely correspond to deactivation of ARF6-GTP. Alternatively, the loss of FRET signal may be attributable to displacement of the YFP-NGAT reporter by endogenous effectors. The activation profile of ARF6 was essentially identical to the profile for Cdc42, which preceded maximal Rac1/Rac2 activation [ 30]. The activation of ARF6 and Cdc42 in a confined band at the leading edge of pseudopods is manifested in the particle-tracking results as initially high FRET values that begin to trend downward immediately due to the advance of the macrophage cytoplasm into the phagosome-measurement region, which displays low levels of activated ARF6 (see Figure 3B). This indicates that Cdc42 and ARF6 regulate processes required for the extension of pseudopods immediately after binding of opsonized targets. The activation profiles for ARF6, Cdc42, and Rac estimated using FRET microscopy and particle-tracking analysis differ from the profiles measured biochemically [ 4]; pull-down assays indicated that Rac activation precedes Cdc42 and ARF6 activation. This difference may be explained by the superior temporal resolution provided by the FRET microscopic approach.

ARF6 activation appeared to be independent of PI-3K activity as LY294002 did not reduce ARF6-GTP levels in the phagocytic cup ( Figure 4A and 4B and Video S4). Rather, PI-3K function was required for the deactivation of ARF6, suggesting that PI-3K regulates closure activities rather than pseudopod extension. The activation of ARF6 was surprising given previous studies that demonstrated GEFs from the ARNO/cytohesin family translocate to the plasma membrane in a PI-3K-dependent fashion [ 38, 39]. Based on our results, we hypothesize that the redistribution of these GEFs in a PI-3K-dependent manner correlates with ARF1, not ARF6, activation, and that ARF1 regulates phagosome closure. Accordingly, ARF1 activation at phagosomes was reduced by LY294002, while activation of ARF1 in the Golgi compartment was unaffected by the presence of the PI-3K inhibitor ( Figure 4C and 4D and Video S5). Also, the peak activation of ARF1 occurred after maximum ARF6 activation and was not confined to the leading edge of pseudopods.

The inability of BFA to inhibit phagosome formation led to the hypothesis that ARF1 activation is not required for phagocytosis [ 3]. However, the possibility of ARF1 activation by BFA-insensitive GEFs during phagocytosis was not excluded. We observed that BFA did not alter the timing or magnitude of ARF1 activation during phagocytosis, suggesting that ARF1 is an in vivo substrate of ARNO/cytohesin-family GEFs. The activation of ARF1 during phagocytosis did not appear to be gratuitous as we observed that ARF1(T31N) and ARF1(Q71L) blocked phagocytosis after the initiation of pseudopod formation. As ARF6 was activated primarily during the early time points of pseudopod extension, it is unlikely that the blockade induced by the ARF1 cycling mutants was due to a nonspecific effect on ARF6 activation. Given the prominent role of ARF1 in the trafficking of proteins through the Golgi network, it is possible that the effects of the ARF1 mutants on phagocytosis are due to a nonspecific effect on receptor trafficking. However, given that wild-type ARF1-CFP also reduced the binding indexes of macrophages without impacting their ability to phagocytose bound targets, it is possible the reduced binding indexes are simply a reflection of the different morphology of cells expressing ARF1-CFP chimeras. Alternatively, the reduction of binding indexes seen in cells expressing the cycling mutant ARF1 chimeras may reflect a role for ARF1 in the trafficking of Fcγ receptors.

The fact that their activation dynamics are independently regulated during phagocytosis suggests that these ARF GTPases coordinate distinct activities at the phagosome. ARF6 appears primarily to control early events in phagocytosis that occur upstream of PI-3K signaling [ 4]. ARF6 may coordinate these processes in conjunction with Cdc42 and Rac1 [ 30], including the membrane delivery and actin polymerization required to envelop target particles. ARF1 on the phagosome is activated downstream of PI-3K amplification of receptor signaling. ARF1 may be involved in the oxidative burst coupled to FcγR-mediated phagocytosis [ 40]. ARF1 was previously implicated in the BFA-resistant, fMLP-stimulated activation of PLD, a process that leads to increased production of reactive oxygen intermediate in neutrophils [ 15]. ARF1 might also stimulate the oxidative burst through its ability to bind Arfaptin. Arfaptin has been shown to bind to Rac proteins in both their GDP- and GTP-bound states but specifically binds to activated ARF GTPases [ 41]. Accordingly, Arfaptin might sequester Rac in the absence of ARF-GTP. Generation of ARF-GTP would free Rac for incorporation into functional phagocyte oxidase complexes. In this model, ARF1 would co-activate the oxidative burst with Rac2, a GTPase that is primarily active during the closure phase of phagocytosis [ 30]. Given the restriction of ARF6 activation to the leading edge of the phagosome and the profile of Rac activation described previously [ 30], ARF1 would appear to be a better candidate for this function.

A crucial question is how the activity of the BFA-resistant GEFs could regulate ARF6 and ARF1 in distinct patterns. Independent regulation of ARF6 and ARF1 may depend on the preference of the GEFs' exchange domains for different ARFs. EFA6 can efficiently catalyze nucleotide exchange on ARF6 but not on ARF1; the constitutive localization of this GEF to the plasma membrane may allow it to function in the absence of PI-3K activity [ 42]. This differs from the recently identified, BFA-resistant GEF cytohesin-4 [ 43]. Cytohesin-4, expressed in leukocytes, was capable of activating ARF1 in vitro but was inactive toward ARF6. Identification of the GEFs responsible for ARF activation during phagocytosis, and determination of their PI-binding preferences could provide insight into the differential regulation of ARF GTPases during phagocytosis.

## Materials and Methods

### Molecular cloning and DNA manipulation.

Plasmids incorporating monomeric versions (A207K) of the GFP spectral variants ECFP and Citrine (an EYFP variant) were used for all constructs [ 44, 45]. Human ARF1 and ARF6 from the UMR cDNA Resource Center ( http://www.cdna.org) were PCR-amplified for subcloning into pECFP-N1 and pEYFP-N1 (Clontech, Mountain View, California, United States) on EcoRI-KpnI fragments producing a fusion to the fluorescent protein at the C-terminus of the GTPase. To generate YFP-NGAT, the base pairs encoding amino acid residues 165–210 from transcript variant 1 of human GGA1, a gift from J. Bonifacino, were PCR-amplified for insertion into the XhoI-EcoRI site of pEYFP-C1. YFP-NGAT was mutated to NGAT(A193T, N194Y) in the YFP-NGAT expression construct using the QuikChange site-directed mutagenesis kit (Stratagene, La Jolla, California, United States). ARF1(T31N), ARF1(Q71L), ARF6(T27N), and ARF6(Q67L) were generated from the appropriate ARF-CFP expression constructs using the Stratagene QuikChange mutagenesis kit. The sequences of constructs were confirmed by DNA sequencing at the University of Michigan DNA Sequencing Core.

### Tissue culture and transfection.

RAW264.7 macrophage-like cells were obtained from the American Type Culture Collection (Manassas, Virginia, United States) and maintained at 37 °C under 5% CO _2_. RAW264.7 cells were cultured in Dulbecco's Modified Eagle Medium (DMEM) supplemented with 10% HIFBS, 100 U/ml penicillin, and 100 μg/ml streptomycin, or Advanced-DMEM supplemented with 2% HIFBS, 4 mM L-glutamine, 20 U/ml penicillin, and 20 μg/ml streptomycin. Cell culture reagents were products of Invitrogen (Carlsbad, California, United States). Cells under microscopic observation were maintained in Leiden chambers (Harvard Apparatus, Holliston, Massachusetts, United States) at 37 °C in Ringer's buffer (155 mM NaCl, 5mM KCl, 2 mM CaCl _2_, 1 mM MgCl _2_, 2 mM NaH _2_PO _4_, 10 mM glucose, 10 mM HEPES [pH 7.2]). The opsonization of sheep erythrocytes with rabbit IgG (ICN Biochemicals, Aurora, Ohio, United States) has been described previously [ 46].

To prepare RAW264.7 cells for FRET or ratiometric microscopy, ˜2.5 × 10 ^5^ cells per coverslip were plated the day before imaging. Transfection of plasmids encoding fluorescent chimeras was performed ˜18 h prior to the start of imaging using FuGene-6 according to the manufacturer's recommended protocol (Roche Diagnostics, Indianapolis, Indiana, United States).

### Pharmacological treatments.

BFA and LY294002 were purchased from EMD Biosciences (San Diego, California, United States). Stocks were prepared by dilution of the lyophilized solids into DMSO to a concentration of 1 mM for BFA and 10 mM for LY294002. Aliquoted stocks were stored at −20 °C until needed. BFA was added to the cells in the Leiden chamber to a final concentration of 5 μM, and fluorescence images were recorded as described below. LY294002 was added to cells at a final concentration of 50 μM 30 min prior to the addition of opsonized erythrocytes.

### FRET microscopy.

FRET component fluorescence images were acquired using a Nikon Eclipse TE-300 inverted microscope with a 60×, numerical aperture 1.4, oil-immersion PlanApo objective lens (Nikon, Tokyo, Japan) and Lambda LS xenon arc lamp for epifluorescence illumination (Sutter Instruments, Novato, California, United States). Image acquisition and processing were performed using Metamorph 6.2r6 (Universal Imaging, Malvern, Pennsylvania, United States). Fluorescence excitation and emission wavelengths were selected using a JP4v2 filter set (Chroma Technology, Rockingham, Vermont, United States) and a Lambda 10–2 filter wheel controller (Sutter Instruments) equipped with a shutter for epifluorescence illumination control. Phase contrast illumination was controlled using a Uniblitz VMM-D1 shutter driver (Vincent Associates, Rochester, New York, United States). Images were recorded with a Photometrics CoolSnap HQ cooled CCD camera (Roper Scientific, Tucson, Arizona, United States). Images were acquired by positioning excitation and emission filters to visualize CFP (I _D_): excitation at 430 ± 12.5 nm, emission at 470 ± 15 nm; YFP (I _A_): excitation at 500 ± 10 nm, emission at 535 ± 15 nm; or FRET (I _F_): excitation at 430 ± 12.5 nm, emission at 535 ± 15 nm. Images were collected with exposure times of 100–400 ms. To account for variable exposure lengths, image scaling was performed following shading and bias correction, e.g., to correct for I _D_:I _F_:I _A_ exposure lengths of 200 ms : 200 ms : 100 ms, the shade/bias corrected I _A_ image was multiplied by two prior to further image processing. Shading correction images for I _D_, I _A_, and I _F_ were collected from a mixture of CFP and YFP between two coverglasses. Bias-correction images to correct for camera dark noise were collected with the excitation light blocked. The FRET parameters *α* and *β* were measured from RAW264.7 cells expressing YFP or CFP, respectively. The parameters *γ* and *ξ* were determined using cells expressing a covalently linked CFP–YFP molecule, whose FRET efficiency (E _C_) has been measured by fluorescence lifetime spectroscopy, and back-calculating using the expression for *f _A_* and an updated expression for *f _D_* (see Protocol S1), respectively [ 24]. The E _A_, E _D_, and R _M_ images were calculated from the corrected fluorescence images and FRET parameters as described here in Protocol S1 (for E _D_ and R _M_) and as described in previous work (E _A_) [ 24].

To observe phagocytosis, RAW264.7 cells expressing fluorescent chimeras were located, and phagocytosis initiated by delivery of ˜2 × 10 ^5^ IgG-opsonized erythrocytes to the chamber. Collection of I _A_, I _D_, I _F_, and phase contrast images began as erythrocytes landed on cells expressing fluorescent chimeras. Image sets were recorded within 2 s at 30-s intervals. Following acquisition of complete time series, shade/bias-corrected I _A_, I _D_, and I _F_ images were used to calculate the FRET stoichiometry images E _A_, E _D_, and R _M_. The calculated images represent: the fraction of YFP chimera *(f _A_)* in complex times the characteristic FRET efficiency (E _C_) of the complex (E _A_), the fraction of CFP chimera in complex *(f _D_)* times E _C_ (E _D_), or the molar ratio of YFP chimera to CFP chimera (R _M_) at each pixel in the image. Note that the value of E _C_ for the ARF–CFP–YFP–NGAT interaction is not required for calculation of E _A_, E _D_, and R _M_.

To measure the BFA-mediated disruption of ARF1 activation at the Golgi complex, I _A_, I _D_, I _F_, and phase contrast images of RAW264.7 macrophages expressing both ARF1-CFP and YFP-NGAT were collected before the addition of BFA (final concentration of 5 μM). Following the addition of toxin, fluorescence and phase contrast images were collected at 30-s intervals. Image processing to obtain the E _A_, E _D_, and R _M_ images was performed as described above. To define the Golgi compartment, a manual threshold was applied to the high intensity region in the shade/bias-corrected I _D_ image. This threshold was used to create a binary mask; the mask was applied to the E _A_, E _D_, and R _M_ images to define the region of measurement. Metamorph's Integrated Morphometry Analysis was used to measure the area and average gray value of the resulting region in the masked E _A_, E _D_, and R _M_ images at each time point. As BFA reduced the Golgi network, the region of high intensity used to make measurements decreased in size. After ˜15–20 min in 50 μM BFA, the measurement region could no longer be defined due to redistribution of ARF1-CFP. Cells expressed uniform E _D_ values of ˜0 at these time points.

To compare FRET values measured in cells transfected with different ARF-CFP and YFP-NGAT chimeras, the average fluorescence intensities of the masked, shade-corrected, and bias-corrected I _A_, I _D_, and I _F_ component images for each cell were recorded into a spreadsheet. The average fluorescence values were then used to calculate E _A_, E _D_, and R _M_ using the FRET stoichiometry equations and E _AVG_, the arithmetic mean of E _A_ and E _D_. Because E _A_ and E _D_ are sensitive to the amount of acceptor and donor available for incorporation into FRET complexes, these values are dependent on the value of R _M_; while E _A_ is suppressed in cells that express a large excess of YFP relative to CFP, E _D_ is increased in cells with an overabundance of acceptor. E _AVG_ serves as a weighted average that adjusts for varying levels of donor and acceptor in a population of cells, and was therefore used to compare FRET values in cells that express variable relative amounts of different CFP and YFP chimeras. The relationships between E _A_, E _D_, E _AVG_, and R _M_ can be seen in Figure S1. Statistical analysis to compare FRET in cells expressing the wild-type and mutant chimeras was performed using Prism 3.0 (GraphPad Software, San Diego, California, United States).

### Ratiometric microscopy.

During phagocytosis by cells expressing YFP chimeras, quantification of the association of the YFP chimera with the phagosome is complicated by the changes in optical path length induced by morphological rearrangements at the cell periphery near the phagosome. Ratiometric imaging can be used to distinguish specific association of a marker with the phagosome from intensity changes due to the increase in bulk cellular material at the site of phagocytosis [ 47, 48]. The application of FRET stoichiometry to ratiometric imaging analysis allows the quantification of the molar ratio of YFP chimera to soluble CFP marker [ 30]. In the absence of CFP–YFP energy transfer, the molar ratio can be calculated as:



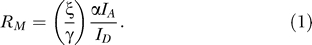



The factors *α* and *γ* are defined as described previously, *ξ* is described in Protocol S1, and we have replaced the notation R with R _M_ [ 24]. To make measurements of protein localization that are independent of the relative transfection efficiency of the YFP chimera and CFP in a particular cell, a recruitment index has been developed [ 30]. This recruitment index is calculated as R _M_ for the phagosome region divided by R _M_ for the entire cell:



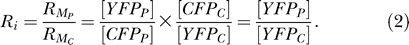



Image collection for ratiometric microscopy was performed as for FRET microscopy except that only the I _A_, I _D_, and phase contrast images were collected.

### Particle-tracking and phagosome analysis.

Methods for measuring phagosome-associated signals using the centroid-tracking algorithm TRACKOBJ in Metamorph have been described [ 30, 48]. Briefly, the TRACKOBJ algorithm was used to identify the center of the erythrocyte in the phase contrast image and to position a 5-μm circular region encompassing the erythrocyte in the phase contrast, E _A_, E _D_, and R _M_ images. The average E _A_, E _D_, and R _M_ values of the phagosome measurement region, excluding the non-cell portion, were then recorded for each image in the series. Phagocytic progress was monitored using the phase-bright to phase-dark transition that accompanies phagocytosis of opsonized erythrocytes [ 49]. Phagosomes were aligned at the time point corresponding to the midpoint of the phase-bright to phase-dark transition; this corresponds to 7.5 min. For phagocytic cups formed in the presence of LY294002, particle-tracking measurements were temporally aligned at the onset of pseudopod extension observed in the fluorescence images.

### Measurement of phagocytic efficiencies.

To quantify the effects of the ARF GTPase mutants on phagocytosis, phagocytic efficiencies were measured for cells expressing the GTPases as CFP fusions. ˜5 × 10 ^4^ RAW264.7 cells were plated in triplicate onto 13-mm coverslips. The cells were transfected the same day with CFP-expression plasmid or plasmids encoding the CFP fusions of ARF1, ARF1(T31N), ARF1(Q71L), ARF6, ARF6(T27N), or ARF6(Q67L). The following day, ˜10 ^6^ IgG-opsonized sheep erythrocytes were delivered to each coverslip in 50 μL of warm, serum-free DMEM. Erythrocytes were allowed to sediment and phagocytosis to proceed for 30 min in a 37 °C–CO _2_ incubator. Phagocytosis was arrested and unbound erythrocytes removed by rinsing the coverslips three times with PBS followed by fixation of cells in 20 mM HEPES, 0.2 M sucrose, 4% PFA, 0.01% glutaraldehyde [pH 7.4] for 30 min; fixation and all subsequent steps were performed at room temperature. Following three rinses with PBS, cells were treated with sodium borohydride (Sigma Aldrich, St. Louis, Missouri, United States) to reduce primary fluorescence induced by glutaraldehyde. Coverslips were incubated with a 1 mg/mL solution of borohydride in PBS for 5 min. After four incubations in the borohydride solution, cells were rinsed six times with PBS and twice with PBS supplemented with 2% goat serum (PBS-GS); coverslips were left in the second PBS-GS rinse for 15 min to block nonspecific binding sites.

To mark uninternalized erythrocytes, coverslips were incubated with Alexa Fluor 488-conjugated goat anti-rabbit IgG (Invitrogen, Carlsbad, California, United States) at a concentration of 1 μg/mL in PBS-GS for 30 min. Cells were rinsed with PBS-GS followed by PBS and mounted on slides using Prolong Gold antifade reagent (Invitrogen, Carlsbad, California, United States).

Visualization of CFP and Alexa Fluor 488 was accomplished on the inverted Nikon Eclipse TE300 FRET microscope using the JP4v2 filter set. Alexa Fluor 488 was imaged through the YFP channel of this filter set. Cells transfected with the CFP expression construct were located on coverslips, and then the internalized erythrocytes (Alexa488 ^−^) and bound, uninternalized erythrocytes (Alexa488 ^+^) counted. The percent phagocytosis was calculated from at least 25 CFP-positive macrophages on each coverslip as:



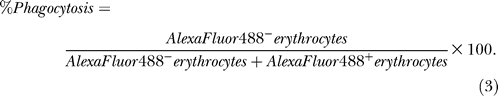



The binding index was calculated as the sum of the uninternalized and internalized erythrocytes per macrophage. Statistical analysis of the phagocytic efficiencies was performed using SigmaPlot 8.0 (Systat Software, Point Richmond, California, United States).

## Supporting Information

Figure S1 FRET Values (E _A_, E _D_, and E _AVG_) Measured from Cells Expressing ARF1-CFP and YFP-NGAT and YFP-Rac2V12 and PBD-CFP, Graphed as Functions of R _M_
The average FRET values were calculated from ˜40 cells expressing ARF1-CFP and YFP-NGAT (A) or ˜125 cells expressing YFP-Rac2V12 and PBD-CFP (B). The construction of the Rac-PBD FRET pair is described elsewhere [ 30]. The values of E _A_, E _D_, and E _AVG_ for each cell were then plotted against the corresponding measured R _M_ values. For R _M_ values below 1, the CFP donor is in excess, while for R _M_ values above 1, the YFP acceptor is the excess binding partner. For low R _M_ values, more acceptors are able to form complex with the excess donors, leading to relatively higher E _A_ values; however, the lack of sufficient acceptors reduces the fraction of donors that can enter into FRET complexes and reduces E _D_. In the case of excess acceptors, the impact on E _A_ and E _D_ values is reversed, with E _A_ being suppressed. E _AVG_, though, does not demonstrate this sharp, asymmetric dependence on R _M_. When graphed as a function of R _M_, E _AVG_ is essentially independent of R _M_, particularly in the range R _M_ = 0.1 – 10. Because E _A_ and E _D_ are distinctly dependent on R _M_, E _AVG_ is preferred for comparing FRET values for multiple samples where average R _M_ values differ between some samples.
(204 KB PDF)

Protocol S1 Explanation of the Changes to the E _D_ and R _M_ Expressions
(25 KB DOC)

Table S1 Table of E _A_, E _D_, E _AVG_, and R _M_ Values from Cells Expressing the ARF-CFP and YFP-NGAT Molecules Measured for Figure 1C
(40 KB DOC)

Video S1 Movie of ARF6 Activation during Phagocytosis A phase contrast movie of an RAW264.7 macrophage engulfing an IgG-coated RBC is shown in the panel to the left. I _A_, I _D_, and I _F_ images were used to calculate the time-lapse R _M_ images (center panel), E _D_ images (right-hand panel) and E _A_ images (unpublished data). Activated ARF6 can be seen at a site of ruffling (upper left of the macrophage, near the center of the panels). Phagocytosis of an erythrocyte, initiated below the ruffle, induced the activation of ARF6 at the leading edge of the phagosome pseudopod during the extension phase. ARF6 was deactivated as the phagosome sealed. All videos seen here play at 6 frames/s. Frames were collected at 30-s intervals.
(2.9 MB MOV)

Video S2 Movie of ARF1 Activation during Phagocytosis Images shown left to right: phase contrast, R _M_ and E _D_ time-lapse images of a macrophage expressing ARF1-CFP and YFP-NGAT. The activation of ARF1-CFP at the phagosome is revealed in the E _D_ image. ARF1 activation at the phagosome increased during the extension phase and was resolved during closure. The juxtanuclear Golgi compartment is visible in the E _D_ image as well, to the lower right of the forming phagosome. Levels of activated ARF1 at the Golgi did not vary during phagocytosis.
(2.2 MB MOV)

Video S3 Movie of ARF1 Activation during Phagocytosis by a BFA-Treated Macrophage Panels in the video, left to right: phase contrast, R _M_ and E _D_ images from a macrophage expressing ARF1-CFP and YFP-NGAT, and treated with 5 μM BFA for 30 min prior to microscopic observation. Exposure to BFA prevented the activation of ARF1 at the Golgi—no regions of high FRET are visible near the nucleus in the E _D_ image at the start of the movie. However, the two phagocytic events (lower right, followed by upper left) led to the activation of ARF1, indicating that ARF1 activation at the phagosome is mediated by a BFA-resistant ARNO/cytohesin-family GEF.
(2.7 MB MOV)

Video S4 Movie of ARF6 Activation in the Presence of LY294002 RAW264.7 macrophages co-transfected with ARF6-CFP and YFP-NGAT were incubated in 50 μM LY294002 for 30 min before the addition of IgG-opsonized erythrocytes. The presented images, left to right, are: phase contrast, R _M_ and E _D_ time-lapse images. Following binding of a particle at the right, ARF6-CFP was activated and pseudopod extension commenced. GTP-bound ARF6-CFP continued to accumulate in the phagocytic cup and was not deactivated during observation. The accumulation of ARF6-CFP at the arrested phagocytic cup can be seen in the R _M_ image series (the blue band at the phagocytic cup represents a molar excess of ARF6-CFP relative to YFP-NGAT). The generation of ARF6-GTP in the absence of PI-3K function indicates ARF6 is activated at the phagosome by a PI-3K-independent GEF activity.
(2.8 MB MOV)

Video S5 Movie of ARF1 Activity with PI-3K Activity Blocked by LY294002 RAW264.7 macrophages co-transfected with ARF1-CFP and YFP-NGAT demonstrated high FRET signals in the area of their Golgi network in the presence of LY294002: the nucleus is visible in the phase contrast image shown at the left; next to the nucleus, the crescent-shaped region of high E _D_ (far-right panel) corresponds to the Golgi apparatus. The Golgi is also visible in the R _M_ image (center panel) where ARF1-CFP is present in excess over YFP-NGAT. Cells treated with LY294002 could bind opsonized particles and form phagocytic cups but could not complete phagocytosis. At the upper left and right of the macrophage, binding of opsonized erythrocytes was not accompanied by the activation of ARF1-CFP. This result indicates that amplification of FcγR-signaling by PI-3K is required for the activation of ARF1 at the phagosome.
(2.9 MB MOV)

### Accession Numbers

Genbank ( http://www.ncbi.nlm.nih.gov/Genbank) accession numbers are: ARF1 cDNA used to produce ARF1-CFP (AF493881), ARF6 source cDNA (AF493885), GGA1 transcript variant used to create NGAT (NM_013365). All plasmids used to produce the data for this manuscript can be obtained from the Addgene plasmid repository ( http://www.addgene.org/Joel_Swanson, and the Addgene plasmid ID numbers are 11381–11390.

## Abbreviations

ARF: ADP ribosylation factor
BFA: brefeldin A
CFP: cyan fluorescent protein
FcγR: Fcγ receptor
FRET: fluorescence resonance energy transfer
GAP: GTPase activating protein
GAT: GGA and TOM
GEF: guanine nucleotide exchange factor
GGA: Golgi-localized
GTP: guanosine 5′-triphosphate
GTPase: GTP hydrolase
NGAT: N-terminal region of GAT
PI: phosphoinositides or phosphatidylinositol
PI-3K: phosphatidylinositol-3′-kinase
PLD: phospholipase D
SEM: standard error of the mean
YFP: yellow fluorescent protein
