# Supplementary material for: A Phosphatidylinositol-3-Kinase-Dependent Signal Transition Regulates ARF1 and ARF6 during Fcγ Receptor-Mediated Phagocytosis
Source: PLoS Biol. 2006 May 9;4(6):e162. doi: 10.1371/journal.pbio.0040162 (PMC1457017; doi:10.1371/journal.pbio.0040162)
Supplement: Supplementary file 1 [file pbio.0040162.sg001.pdf]

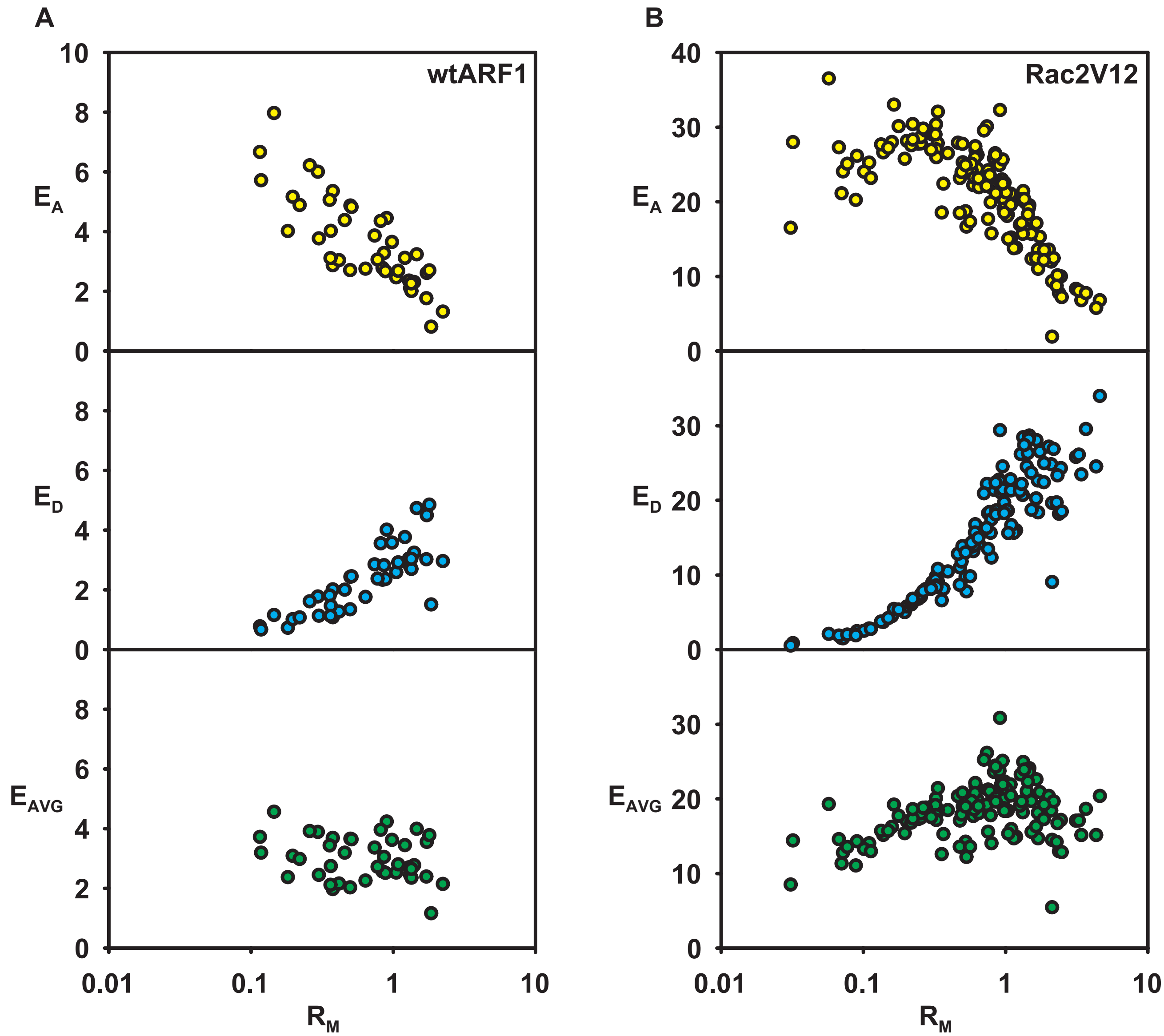

A
B
wtARF1
Rac2V12
E_A
E_D
E_AVG
R_M
10
8
6
4
2
0
40
30
20
10
0.01
0.1
1
10
