# Supplementary material for: A Phosphatidylinositol-3-Kinase-Dependent Signal Transition Regulates ARF1 and ARF6 during Fcγ Receptor-Mediated Phagocytosis
Source: PLoS Biol. 2006 May 9;4(6):e162. doi: 10.1371/journal.pbio.0040162 (PMC1457017; doi:10.1371/journal.pbio.0040162)
Supplement: Supplementary file 2 [file pbio.0040162.sd001.doc]

**Protocol S1. Supporting Equations. Explanation of changes to E_D_ and R_M_.**

For FRET stoichiometry, the total fluorescence of the CFP donor corrected for energy transfer to YFP can be expressed as:

$F_{D}\left(corrected for FRET\right)=\xi\times F_{ET}+F_{DA}$.

Where *F_ET_* is the sensitized emission of the acceptor (YFP fluorescence due to energy transfer), and *F_DA_* is the fluorescence of the donor in the presence of acceptor. Multiplication of *F_ET_* by *ξ* determines the CFP fluorescence lost due to energy transfer to YFP. This differs from our previous expression for the total fluorescence from CFP in that the sensitized emission is now multiplied by *ξ*, rather than *ξ/γ* [30]. As the sensitized emission of YFP arises from photons that are absorbed by CFP, it is unnecessary to divide by *γ*, which corrects for the difference in photon absorption of YFP and CFP at the CFP excitation wavelength. Substituting this expression into the FRET stoichiometry equation, E_D_ can be written as:

$E_{D}=\left[1-\frac{I_{D}}{\left(I_{F}-\alpha I_{A}-\beta I_{D}\right)\xi+I_{D}}\right]$.

Similarly, the expression for R_M_ can now be written as:

$R_{M}=\frac{\left[Total Acceptors\right]}{\left[Total Donors\right]}=\left(\frac{\xi}{\gamma}\right)\frac{\alpha I_{A}}{\left(I_{F}-\alpha I_{A}-\beta I_{D}\right)\xi+I_{D}}$.
