# Supplementary material for: A Phosphatidylinositol-3-Kinase-Dependent Signal Transition Regulates ARF1 and ARF6 during Fcγ Receptor-Mediated Phagocytosis
Source: PLoS Biol. 2006 May 9;4(6):e162. doi: 10.1371/journal.pbio.0040162 (PMC1457017; doi:10.1371/journal.pbio.0040162)
Supplement: Supplementary file 3 [file pbio.0040162.st001.doc]

**Table S1.** **E_A_, E_D_, E_AVG_ and R_M_ values for Figure 1C.**

| **wildtype NGAT** | **E_A_±σ** | **E_D_±σ** | **R_M_±σ** | **E_AVG_±σ** |
| --- | --- | --- | --- | --- |
| **ARF1** | 3.65±1.52 | 2.34±1.11 | 0.82±0.56 | 3.00±0.74 |
| **ARF1 (Golgi)** | 8.02±3.88 | 4.17±2.48 | 0.71±0.77 | 6.09±2.19 |
| **ARF1(T31N)** | 1.21±1.39 | 0.69±0.89 | 0.98±0.87 | 0.95±0.96 |
| **ARF1(Q71L)** | 5.50±2.97 | 3.71±1.69 | 0.89±0.82 | 4.60±2.07 |
| **ARF1(Q71L) (Golgi)** | 9.45±3.76 | 5.39±2.15 | 0.66±0.52 | 7.42±2.41 |
| **ARF6** | 1.74±0.96 | 1.15±0.56 | 0.84±0.56 | 1.45±0.66 |
| **ARF6(T27N)** | 0.44±0.36 | 0.58±0.45 | 1.93±0.92 | 0.51±0.38 |
| **ARF6(Q67L)** | 5.70±2.45 | 3.87±1.21 | 0.80±0.38 | 4.78±1.53 |

| **NGAT(TY)** | **E_A_±σ** | **E_D_±σ** | **R_M_±σ** | **E_AVG_±σ** |
| --- | --- | --- | --- | --- |
| **ARF1** | 1.63±1.43 | 0.46±0.32 | 0.36±0.31 | 1.04±0.79 |
| **ARF1 (Golgi)** | 2.07±3.85 | 0.19±0.18 | 0.19±0.24 | 1.13±1.99 |
| **ARF1(Q71L)** | 1.35±1.09 | 0.38±0.54 | 0.60±1.0 | 0.86±0.70 |
| **ARF1(Q71L) Golgi** | 1.51±2.43 | 0.13±0.51 | 0.40±1.06 | 0.82±1.34 |
| **ARF6** | 1.14±0.87 | 0.70±0.65 | 0.76±0.62 | 0.92±0.69 |
| **ARF6(Q67L)** | 2.45±1.99 | 0.53±0.39 | 0.28±0.20 | 1.49±1.13 |
